# Lessons Learned During the COVID-19 Pandemic to Strengthen TB Infection Control: A Rapid Review

**DOI:** 10.9745/GHSP-D-21-00368

**Published:** 2021-12-31

**Authors:** Helena J. Chapman, Bienvenido A. Veras-Estévez

**Affiliations:** a Department of Environmental and Occupational Health, Milken Institute School of Public Health, George Washington University, Washington, DC, USA.; b Facultad de Ciencias de la Salud, Universidad Católica del Cibao, La Vega, Dominican Republic.

## Abstract

**Introduction:**

Over the past 5 years, substantial global investment has resulted in reduced TB incidence rates by 9% and mortality rates by 14%. However, the coronavirus disease (COVID-19) pandemic has hindered access and availability of TB services to maintain robust TB control. The objective of this rapid review was to describe the challenges to be addressed and recommendations to strengthen health system preparedness for optimal TB control across low- and middle-income countries during and after the COVID-19 pandemic.

**Methods:**

Five databases were used to systematically search for relevant articles published in 2020. The 5-step framework proposed by Arskey and O'Malley and adapted by Levac et al. guided the review process. Thematic analysis with grounded theory principles was used to summarize themes from selected articles and integrate analyses with barriers reported from authors' previous TB research.

**Results:**

Of the 218 peer-reviewed articles, 20 articles met the inclusion criteria. Four emerging themes described challenges: (1) unprepared health system leadership and infrastructure, (2) coexisting health priorities, (3) insufficient health care workforce support for continued training and appropriate workplace environments, and (4) weak connections to primary health centers hindering community engagement. Four recommendations were highlighted: (1) ensuring leadership and governance for sustainable national health budgets, (2) building networks of community stakeholders, (3) supporting health care workforce training and safe workplace environments, and (4) using digital health interventions for TB care.

**Conclusions:**

National health systems must promote patient-centered TB care, implement ethical community interventions, support operational research, and integrate appropriate eHealth applications. TB program managers and primary care practitioners can serve as instrumental leaders and patient advocates to deliver high-quality and sustainable TB care that leads to achieving the targets of the End TB Strategy.

## Abstract (precis)

In light of competing health priorities of COVID-19 and TB, we propose recommendations to strengthen health system preparedness for optimal TB control across low- and middle-income countries during and after the COVID-19 pandemic.

## Teaser

**Key Findings:**

Current challenges in TB control efforts, such as an unprepared public health system leadership and infrastructure and an overwhelmed health care workforce, will require novel approaches, practices, and renewed political commitment to maintain sustainable TB programs.

We described proposed recommendations that may strengthen health system preparedness for optimal TB control across low- and middle-income countries:

- Ensuring leadership and governance for sustainable national health care budgets
- Building networks of community stakeholders
- Supporting high-quality health care workforce training and safe workplace environments
- Using digital health interventions for TB care

**Key Implications:**

- National health systems should develop national policies that promote integral, patient-centered TB care, facilitate the implementation of ethical community interventions, support operational research, and allow the integration of appropriate eHealth applications.
- Program managers who understand challenges in TB prevention and control with coexisting health priorities can serve as instrumental leaders and patient advocates to deliver high-quality and sustainable TB care that leads to achieving targets of the End TB Strategy.

## INTRODUCTION

Of respiratory infections, TB is the leading cause of global morbidity and mortality, causing 10 million new TB cases and 1.4 million TB deaths in 2019.^1^ TB is spread through aerosol droplets, including those smaller than 5 microns, infected with *M. tuberculosis* from an infected individual to a susceptible individual. One-third of the global population, which is estimated to have an asymptomatic *M. tuberculosis* infection, has a 5%–10% risk of developing TB disease during their lifespan.^2^ With substantial global investment and political commitment over the past 5 years, TB incidence and mortality rates have continued to decrease by 9% and 14%, respectively.^1^ To minimize disease transmission, strict adherence to evidence-based infection control and prevention measures are recommended in clinical and community settings.^3^

The End TB Strategy has set ambitious milestones for 2025 and targets to end TB by 2035.^4^ Three pillars place precedence on expanding patient-centered TB prevention and control efforts, forming policies and multisector collaborations across communities and public and private sectors, and ensuring continued attention on scientific research innovations for TB care.^4^ However, these robust efforts have been redirected to support the coronavirus disease (COVID-19) response measures. Economic and human resources for health have been diverted to emergency care and contact tracing of COVID-19 patients, and laboratories have been repurposed for COVID-19 diagnostic testing.^5^ As a result, health care services for infectious diseases and comorbidities of significant burden—like TB, HIV/AIDS, and malaria—diminished,^5^ and citizens were fearful to seek TB diagnostic and treatment services.^6^ During the Ebola virus disease outbreak in West Africa in 2014–2015, similar observations were reported, such as reductions in TB, HIV, and malaria case notifications and treatment, reductions in health care utilization including vaccination coverage, and the breakdown of community cohesion due to fear, apprehension, and socioeconomic impacts.^7^^–^^9^

Health systems should recognize the syndemic effects of TB and COVID-19 and analyze 3 factors for best clinical and community management practices of competing health priorities. First, the social determinants of health draw attention to the impact of social environments (e.g., education and income), physical environments (e.g., residence and transportation), and access and quality of health care services, including access to broadband internet.^10^^,^^11^ Second, the One Health concept promotes the development of multidisciplinary collaborations to advance clinical and research applications focusing on the human-animal-environment nexus.^12^^,^^13^ Third, the “knowledge-action” gap can result from limitations in the access of up-to-date scientific knowledge (e.g., health care professions curricula and continuing education), health system infrastructure (e.g., financial resources and surveillance programs), and social environments (e.g., access to health care services and influence of news sources).^14^^,^^15^ Hence, the primary health care workforce can contribute to public debates and community campaigns, develop patient education materials on pressing health issues,^16^ and lead efforts to implement evidence-based findings that can optimize clinical, educational, and research practices related to TB care.

Health systems should recognize the syndemic effects of TB and COVID-19 and analyze 3 factors for best clinical and community management practices of competing health priorities.

To date, numerous countries have reported reduced TB case notifications during the COVID-19 pandemic, reflecting the importance of “The Clock is Ticking” theme for World TB Day 2021.^17^^,^^18^ Over the next 5 years, modeling estimates depict that countries with high TB burden will report up to 20% increased TB mortality or 1.4 million TB deaths,^17^^,^^19^ which will negatively impact the End TB Strategy timeline.^20^ In this article, we summarize encountered challenges to be addressed and proposed recommendations from the literature that can strengthen TB prevention and control efforts during and after the COVID-19 pandemic. Moving forward, national health systems can integrate these recommendations into future practices and policies that strengthen health care service delivery, health care professions education, and research capacity related to TB care.

## METHODS

A rapid review was conducted to review the current literature, identify gaps, and synthesize findings,^21^^,^^22^ related to challenges and recommendations for TB prevention and control in low- and middle-income countries. We used the 5-step framework proposed by Levac et al.,^23^ which was adapted from Arksey and O'Malley,^24^ in our search strategy: (1) identify the research question; (2) identify relevant studies; (3) select studies, (4) chart the data; and (5) collate, summarize, and report the results.^23^^,^^24^ The research questions were: (1) What are the existing challenges in TB prevention and control efforts during the COVID-19 pandemic? and (2) What are the proposed recommendations that can strengthen TB prevention and control efforts during and after the COVID-19 pandemic? This search was simultaneously integrated with findings reported from a previous qualitative study that examined challenges in TB prevention and control in a middle-income country, the Dominican Republic.^25^^–^^27^

### Search Strategy and Selection Criteria

On January 10, 2021, 2 authors (HJC, BAV) searched 5 databases (PubMed, Scopus, Web of Science, EBSCO Academic Search Premier, SciELO Citation Index). They used the terms “COVID-19,” “coronavirus,” “health systems,” and “tuberculosis,” to include original articles, reviews, commentaries, and letters, published between January 1, 2020, and December 31, 2020. After removing duplicate articles, authors independently reviewed each abstract and included articles that described the impacts of TB control efforts during the COVID-19 pandemic and proposed recommendations to strengthen health system efforts for TB control. If any disagreement occurred, authors discussed the abstract until consensus was finalized. Articles written in languages other than English were not excluded.

**FIGURE 1:**
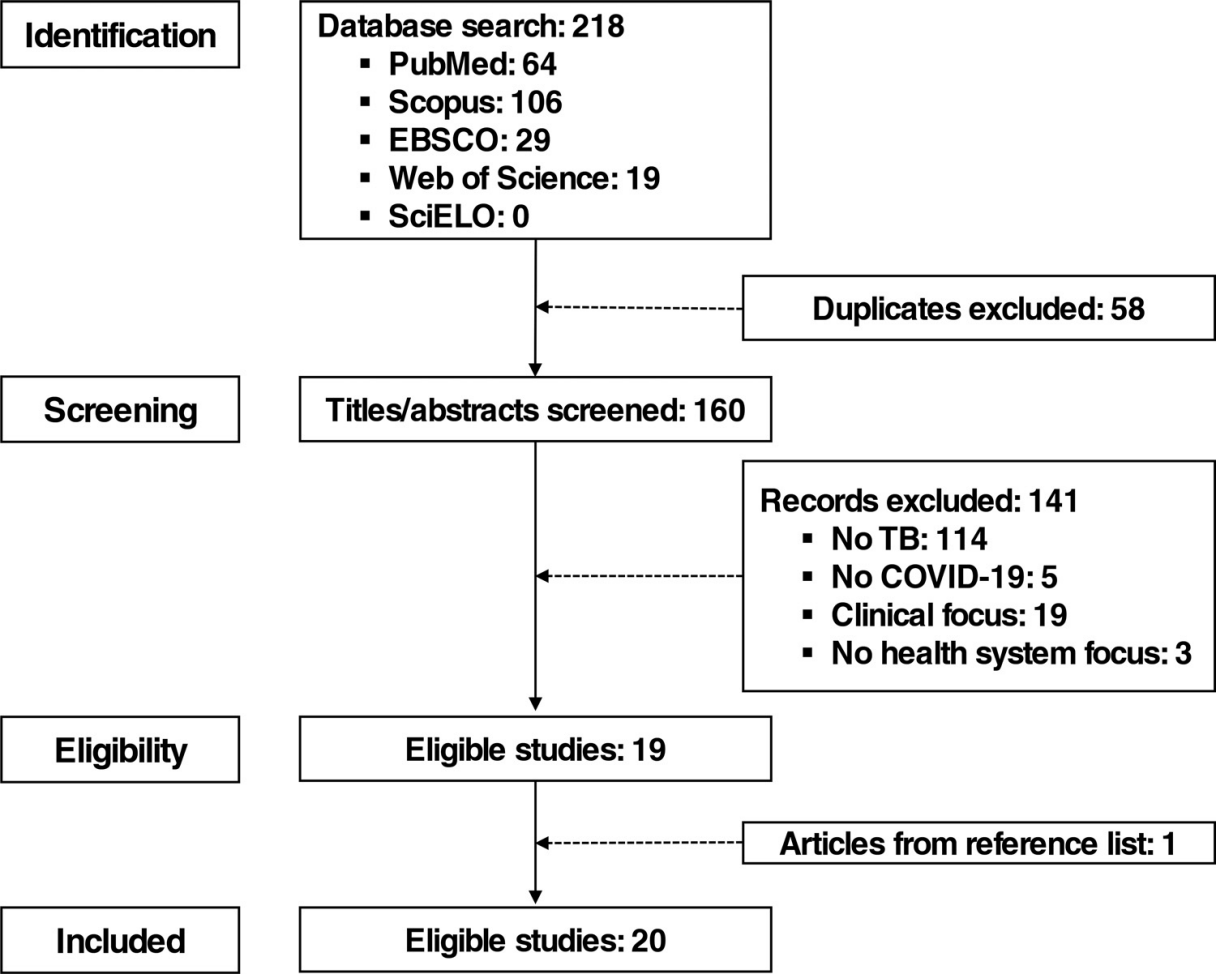
Article Selection Process for a Rapid Review on TB Control Efforts During and After the COVID-19 Pandemic

Articles were excluded if they did not include content related to TB and COVID-19, if clinical aspects of TB and COVID-19 management were described or if the focus deviated from the impact of COVID-19 on TB prevention and control efforts of the health system. Authors incorporated relevant citations from selected articles in the analysis. The article screening and selection process is shown in Figure 1.

### Data Extraction and Analysis

After full-text review, authors used the matrix method, with paper trail, documents, review matrix, and synthesis folders, to extract data from each article.^28^ Data included the country or continent focus, study objective, and main summary points related to challenges to overcome and recommendations for TB prevention and control.

After compiling this information, authors used a qualitative thematic analysis applying grounded theory principles^29^ to identify themes or patterns from selected articles and explore the connections between existing challenges and proposed recommendations to improve TB prevention and control. The 6-step approach of Braun and Clarke^30^ guided the analysis, where authors expanded upon the final themes identified from previous TB research to examine health care workers' perceived barriers to adherence to TB prevention measures in the Dominican Republic.^25^^–^^27^ Authors independently reviewed the initial codes and extracted information to support and expand their meaning. They jointly reviewed and agreed on all final codes, compiled them into relevant themes, and developed a conceptual model.

## RESULTS

**FIGURE 2:**
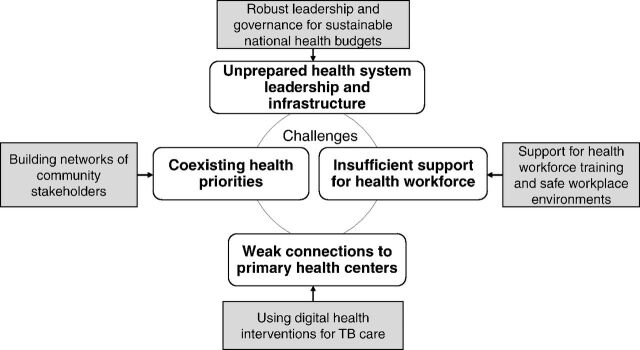
Conceptual Figure That Incorporates Recommendations to Mitigate Existing Challenges and Improve TB Control

The search yielded 218 articles, and after 58 duplicate articles were removed, 160 titles and abstracts were reviewed by both authors. Of the 160 articles, 19 articles met the inclusion criteria, and 1 additional article was added from the manual reference search of these articles. Of the 20 included articles, articles described the TB burden in Africa (n=8), Asia (n=2), and the world (n=10). Article types included 4 letters, 9 perspectives, 5 reviews, and 2 original research articles. Emerging themes from selected articles were identified as existing challenges to be addressed and proposed recommendations to strengthen TB prevention and control efforts (Figure 2).

The characteristics of each article, including proposed recommendations to strengthen TB control efforts during and after the COVID-19 pandemic, are shown (Table 1 and Supplement).

**TABLE 1.** Selected Articles for the Rapid Review of Literature on Recommendations for TB Prevention and Control During the COVID-19 Pandemic in Low- and Middle-Income Countries

Authors	Country or Continent	Recommendations
Togun et al.^6^	UK, Africa	Noting differences between low- and high-income countries in TB priorities (active vs. latent TB), provision of health care services, and mechanisms for social protection, the global response should be comprehensive and long-term, increasing investments in research, innovative digital technology, and public health.
Hogan et al.^19^	Global	The most significant impact to increased mortality was the interruption to antiretroviral therapy for HIV, reductions in timely diagnosis and treatment of new cases for TB, and the interruption of planned net campaigns for malaria.
Visca et al.^20^	Global	With expected increases in TB incidence and mortality, ensuring high levels of adherence to TB treatment through digital innovation can minimize the burden on patients and the health care workforce.
Adamu et al.^31^	Africa	COVID-19 response strategies should shift from isolated programs to integrated health system interventions that are connected with existing programs in public and private sectors.
Alene et al.^32^	Global	Health systems should aim to maintain routine TB services during the COVID-19 pandemic and hence mitigate the impact of COVID-19 on TB prevention and control programs.
Amimo et al.^33^	Africa	Appropriate economic and epidemiological considerations are required to minimize hardships faced by vulnerable populations to access essential health care services for COVID-19 and other epidemic diseases.
Bhargava and Shewade^34^	India	Federal support is required to improve economic and nutrition livelihood through cash transfers, public distribution system of food, and high-quality community TB surveillance and clinical management.
Bulled and Singer^35^	South Africa	International cooperation and country-specific efforts that reflect local resources and needs are required to overcome significant public health risks by the COVID-19 pandemic.
Dara et al.^36^	Global	Complementary COVID-19 and TB responses, including capacity building, active surveillance and monitoring systems, and sustainable economic investment, have the potential to curb disease transmission. People-centered care models, digital technologies, and community-based services can be adapted for the COVID-19 and TB epidemics.
Homolka et al.^37^	Global	TB diagnostic and research infrastructures can be leveraged for SARS-CoV-2 testing and sequencing to examine virus evolution and diversity. High-quality management principles for TB/SARS-CoV-2 diagnostic testing must be followed to ensure validity, reduce biosafety hazards, and support TB diagnostic services.
Jain et al.^38^	India	Restructuring services—such as multimonth dispensing, video-supported therapy, and community-based services—can strengthen TB programs.
Loveday et al.^39^	South Africa	Integrating COVID-19 systems to support TB prevention and control can include developing a platform for public engagement on disease monitoring, strengthening contact tracing with GIS mapping, offering mobile- or video-supported counseling and clinical management, improving health information and surveillance systems, and increasing federal investment.
McQuaid et al.^40^	Global	To ensure continued access to person-centered TB care, sustainable funding, innovative digital technology, and robust community-based surveillance activities can be expanded to reduce the TB and COVID-19 burden.
Mohammed et al.^41^	Ethiopia	Continued investment in TB care and research activities is key to minimizing disruptions to health and research services.
Mukwenha et al.^42^	Zimbabwe	Through collaborations with local and international partners, Zimbabwe leaders can strengthen HIV/TB services by ensuring stockpile availability of diagnostic testing, disseminating accurate health information to TB patients, and adopting real-time surveillance systems.
Papadimos et al.^43^	Global	Deploying point-of-care diagnostics and focusing on telemedicine platforms (albeit challenges like suboptimal internet connectivity or insufficient encryption) have the potential to enhance screening efforts and prevent excess TB mortality.
Sandy et al.^44^	Zimbabwe	Urgent responses include increased funding for equipment (PPE, sputum containers), monthly medication supplies, and integrated TB/HIV programs that distribute appropriate health information.
Saunders and Evans^45^	Global	Integrated health care for TB and COVID-19, research investment, community mobilization, TB-specific social protection, and innovative digital technologies can strengthen TB control efforts during the COVID-19 pandemic.
Zachariah et al.^46^	Global	Skill-building trainings can support outbreak responses (from data collection to scientific writing) and surveillance programs. Investments in health research can strengthen health system resiliency with robust surveillance programs and a prepared workforce.
Zhou et al.^47^	South Africa	Services implemented during the COVID-19 pandemic, such as GIS mapping, can be repurposed to strengthen TB control efforts. Reliable health care services for TB and COVID-19 patients are key to reducing stigma and building trust in health systems.

Abbreviations: COVID-19, coronavirus disease; GIS, geographic information systems; LMIC, low- and middle-income countries; PPE, personal protective equipment; SARS-CoV-2, severe acute respiratory syndrome coronavirus 2.

### Existing Challenges in TB Prevention and Control

Four emerging themes on existing challenges in TB prevention and control efforts are displayed (Table 2).

**TABLE 2.** Four Emerging Themes on Existing Challenges in TB Prevention and Control Efforts

Theme Description	Quote	Stakeholder Perspective
Unprepared health system leadership and infrastructure	National leadership with a limited short-term plan: …*in times of outbreaks, most leaders tend towards control as a style of action together with the temporality of change perceived as short-lived shocks*^31^	Political leadership
	Limited sustainable funding for high burden diseases: *The other challenge being faced is limited funds for HIV and TB programmes due to poor funding by government*^42^ *…the current pandemic has put significant competitive pressure on research and development in other infectious disease areas*^43^	Political leadership, research funding agencies, philanthropic trusts and foundations
	Foresight to identify factors that hinder TB control efforts: *Taken together, the social, economic and biomedical consequences of the COVID-19 pandemic are likely to combine to create a perfect storm with respect to [TB]*^45^	Political leadership, public health leadership, health and social science researchers
Coexisting health priorities	Impact of disruptions on routine TB care: *With the disruptions and reorganization of services in response to the pandemic, delays in TB diagnosis and treatment initiation may result in increased transmission and new cases*^39^	Public health leadership, health care workers
	Lack of understanding of impact of national emergencies on other established health programs: *Given the severity of projections, hospitals across the globe are creating additional critical care surge capacity and limiting patient routine access to care for other diseases like [TB]*^41^	Public health leadership, health care workers
	Limited oversight of the importance of TB programs: …*health service and political leadership, the media and the public focusing on pandemic management and response with limited oversight and accountability of TB programmes*^32^	Public health leadership, health care workers, media sources
Insufficient health care workforce support for training and appropriate workplace environments	Demanding schedule of the health care workforce: *Health workers have been reassigned to meet the COVID-19 testing demand, leading to very few people conducting HIV and TB testing. Medical staff anxiety and burnout is also playing a role in testing, as staff are overwhelmed with COVID-19 testing*^42^	Public health leadership, health care workers
	Health care workforce reassigned to meet national needs: *In many countries, pulmonologists, and infectious disease and public health experts (those also involved in [TB] prevention and care) together with ICU specialists are or have been re-deployed to the frontline to fight COVID-19*^20^	Public health leadership, health care workers
	Lack of sustainable investment in the health care workforce for clinical and research training: *Investing in people and in research training ahead of public health emergencies generates downstream dividends by strengthening health system resilience for tackling pandemics*^46^	Public health leadership, health care workers, academic institutions
Weak connections to primary health centers hindering community engagement	Interruptions of community-based programs hinder patient-provider interactions related to routine health care services: *Although the actual effects on disease programmes remain unclear, some community-based programmes are already being scaled back and experience in high-income settings has shown a substantial reduction in engagement with regular medical care during recent periods of high health system demand*^19^	Public health leadership, health care workers, community leadership, patients
	Community-based programs link citizens to early TB diagnostics and adherence to management: *Many low-income and middle-income countries have high burdens of these three diseases, and millions of people depend on large-scale programmes to control and treat them…Interruptions to control programmes could result in major setbacks, compounding the direct impact of COVID-19*^19^	Public health leadership, health care workers, community leadership, patients

Abbreviation: COVID-19, coronavirus disease.

1. An unprepared public health system leadership and infrastructure was described by limited short-term plans, inadequate funding for high-burden diseases, and limited foresight to identify key factors that hinder TB control efforts.
2. Coexisting health priorities were exacerbated with disruptions in routine TB care, limited understanding of how natural emergencies impact established health programs, and lack of oversight on the importance of TB programs.
3. Insufficient health care workforce support for continued training and appropriate workplace environments was impacted by the demanding schedules of health care workers and the need for reassigned roles to meet national requests.
4. Weak connections to primary health centers resulted in interrupted community-based programs that hindered patient-provider interactions on routine health care services and negatively impacted early diagnostics and adherence to management.

Coexisting health priorities were exacerbated with disruptions in routine TB care, limited understanding of how natural emergencies impact established health programs, and lack of oversight on the importance of TB programs.

### Proposed Recommendations to Strengthen TB Prevention and Control Efforts

Emerging themes on proposed recommendations to strengthen TB prevention and control efforts are listed (Table 3).

**TABLE 3.** Four Emerging Themes on Recommendations to Strengthen TB Prevention and Control Efforts

Recommendation	Quote	Stakeholder Perspective
Ensuring leadership and governance for sustainable national health budgets	Investing in research: *Research, guidance and funding are urgently required to identify, prioritise and deliver those interventions that could best alleviate the impact of COVID-19-related disruptions*^40^ …*building upstream operational research capacity has generated downstream dividends in strengthening health system resilience for tackling pandemics*^46^	Political leadership, public health leadership, research funding agencies, philanthropic trusts and foundations
	Prioritizing community TB care: *Decentralise TB treatment to community health workers and increase access to TB treatment for home-based TB care… support private hospitals, and academic or research centres, to provide TB testing and treatment*^32^	Political leadership, public health leadership, community leadership
	Renewing political will to support existing and emerging health priorities: *…strong political will and support for research communities are essential, especially in low- and middle-income settings, to advocate for and allocate resources needed to investigate these coinciding pandemics*^37^	Political leadership, public health leadership, research funding agencies, philanthropic trusts and foundations
	Supporting social protection of all citizens: *Provision should also be made for [TB]-specific social protection, which could take the form of cash transfers or food parcels for [TB]-affected households*^45^ *This [restoring routine TB services] has to be followed by a combination of measures: access to food through universal PDS, direct cash transfers and making gainful employment available*^34^	Political leadership, public health leadership, community leadership
	Promoting national leadership: *…it is critically important that political leaders possess appropriate cognitive abilities, procedural hard stops, and advisory capacity to put into place effective solutions*^43^	Political leadership
Building networks of community stakeholders	Promoting shared learning among community stakeholders: *A positive aspect to these two pandemics colliding is that people—communities, public health professionals and policy makers—can learn from each other*^6^	Public health leadership, health care workers, community leadership, policy makers
	Forming multi-sectoral community partnerships: *Non-governmental organisations may partner with governments and national [TB] programmes to mitigate the effects of the COVID-19 pandemic on the provision of biomedical care for [TB]-affected households. This might include sharing diagnostic and laboratory capacity and strengthening caregiver and community health worker roles to support care delivery*^45^	Public health leadership, health care workers, community leadership, non-governmental organizations
	Establishing key connections with researchers: *However, as is the case for the capacity of TB diagnostic services, careful planning and close collaboration between the TB, HIV, and COVID-19 research communities will be crucial not to overburden these infrastructures, especially in resource-poor settings*^37^	Community leadership, academic institutions
	Integrating efforts to build trust and reduce stigma in health system efforts: …*it is therefore important to establish reliable health services and strategies that prioritise care for both TB and COVID-19 patients. This approach will assist in building trust in the health system and allow people to take meaningful measures to keep themselves and their families safe*^47^	Public health leadership, community leadership, patients
Supporting high-quality health care workforce training and safe workplace environments	Reinforcing clinical training through short courses: *Provide short-term training for students and health professionals and recruit additional staff to work on TB programs*^32^	Public health leadership, health care workers, academic institutions
	Building research capacity: *SORT IT teaches multiple and practical skills for activities such as generating and utilizing data, conducting operational research and using evidence to influence policy and/or practice*^46^ *Moreover, with a renewed global focus on active case-finding in TB programs, resources dedicated for COVID-19 community-based research, such as household contact tracing or seroprevalence surveys, could easily be linked to programs to test for TB as well, providing a gateway for training, capacity building, and future TB research*^37^	Public health leadership, health care workers
	Ensuring that health care workers are protected in the workplace environment: *In both diseases, the frontline health care workers need to be well trained, equipped, protected, supported and enabled to care for their patients*^36^	Public health leadership, health care workers
	Highlighting health care workers' role in health education: *Health education has the potential mitigating stigmatization. Thus, a unique health education platform that connects the two diseases is strongly needed*.^41^	Public health leadership, health care workers
Using digital health interventions for TB care	Supporting person-centered care model for TB management: *People-centred models of care, including hospitalization for those with severe diseases, community-based services, video-supported treatment or home-based care are among the interventions that can be expanded or adapted for COVID-19*^36^	Public health leadership, health care workers, information technology, patients
	Advancing current technologies to support TB patients in long-term management: *The creation of these systems [at-home management with toll-free helplines and WhatsApp support groups] provides a long-overdue opportunity to replace TB DOT with virtual supportive adherence counselling and clinical management, and could supplement the existing digital and mobile health technologies (mHealth solutions)*^39^	Public health leadership, health care workers, information technology, patients
	Applying novel technology for COVID-19 to enhance TB services: …*many new initiatives that have already arisen from the COVID-19 pandemic, be it in modelling, artificial intelligence for clinical algorithms to predict disease severity, international clinical trial platforms, or drug and vaccine developments*^6^	Public health leadership, health care workers, biomedical scientists, patients
	Using innovative approaches to strengthen TB contact tracing and case notification: *If TB was integrated into the geospatial mapping system set up to inform the COVID-19 tracing efforts and tracking in real time, this could strengthen the tracing of contacts, recording and notification processes of both the TB and RR-TB programmes*^39^	Public health leadership, health care workers, geospatial experts, patients
	Offering additional support for health care workers: *COVID-19 is radically changing the way we manage TB in the immediate future and is forcing us to accelerate the adoption of digital innovations that simplify and facilitate the workload of healthcare workers*^20^	Public health leadership, health care workers, information technology

Abbreviations: COVID-19, coronavirus disease; DOT, directly observed therapy; RR-TB, rifampicin-resistant TB.

#### Ensuring Leadership and Governance for Sustainable National Health Budgets

With the risk of national emergency scenarios—whether natural disasters, infectious disease outbreaks, or conflict—nations should implement policies and strategies and allocate funding that strengthens prevention and mitigation efforts for infectious and chronic diseases.^48^^,^^52^^,^^53^ Political commitment is essential to support core health care services that sustain high-quality point-of-care diagnostics and treatment plans for TB patients as well as balancing these needs with the coexisting pandemic.^19^^,^^37^^,^^41^^–^^43^ These actions can focus on long-term and comprehensive care for TB and coexisting priorities like COVID-19.^6^^,^^32^^,^^36^^,^^45^

Following the World Health Organization's call that all nations should conduct and utilize research capacity, researchers can investigate key scientific questions raised during coinciding pandemics.^37^^,^^41^^,^^46^ For example, operational research should be conducted to examine the influence of social determinants of health on TB and COVID-19, vaccine effectiveness and community acceptability, and adherence to recommended pharmaceutical regimens.^39^^,^^40^^,^^45^^,^^50^ These research findings can inform national health priorities, which can provide a framework for the appropriate allocation of economic resources. Supported by appropriate legislature, authorities can recommend actions for the health sector to best distribute resources for current health priorities and unanticipated emergency scenarios.^50^

#### Building Networks of Stakeholders to Sustain Community Resources

Forming a network of stakeholders—or community groups comprised of individuals who represent different disciplines but share common goals^51^—can help identify community needs and health system vulnerabilities related to TB prevention and control. As community stakeholders share approaches and lessons learned, a multisectoral response that aims to reduce TB burden can drive community engagement with families^6^^,^^31^^,^^43^^,^^49^ and support health care workers in facilitating educational activities as a platform to increase TB awareness and reduce stigma.^41^^,^^45^ By working with community stakeholders, health leaders can identify high-risk communities of poor health status, including inadequate nutrition, overcrowded living conditions, and unemployment, and advocate for creative solutions to improve public welfare programs.^34^^,^^35^^,^^38^ These programs can drive national action to offer social protection for TB patients, such as cash transfers or food parcels, and essential psychosocial support.^45^

Coupled with these health promotion activities, community-based research can highlight the use of key epidemiology tools and trends to better understand the transmission of emerging diseases like COVID-19 and emphasize the existing syndemic and closely linked influencing factors like poverty.^45^ This insight can be applied to current and future plans of TB programs, especially for the diagnosis and management of active and reactivation TB cases.^32^ Health authorities can also streamline public health efforts in COVID-19 and TB control by minimizing duplicated or non-essential approaches and guiding simultaneous surveillance services for rapid response.^37^^,^^49^ By identifying potential funding partners in the public and private sectors, additional financial resources and equipment can be distributed to health facilities.^31^^,^^44^

Community-based research can highlight the use of key epidemiology tools and trends to better understand the transmission of emerging diseases like COVID-19 and emphasize the existing syndemic.

#### Supporting High-Quality Health care Workforce Training and Safe Workplace Environments

A competent health care workforce must be prepared with appropriate knowledge and skills to simultaneously manage endemic and emerging health threats. Health care workers should receive adequate training, appropriate incentives to provide care, and mental health and psychosocial support.^36^^,^^41^ Continuing education programs can offer up-to-date information about clinical guidelines, best practices, public health principles, and timely health topics.^32^ This information can offer insight on the influence of social determinants on TB and COVID-19 patients, especially the impact of co-morbidities (e.g., diabetes mellitus), environmental contamination (e.g., air pollution), and economic hardships (e.g., poverty and overcrowded living conditions), and strategies to reduce stigma and discrimination.^6^^,^^45^ Community fora can offer public platforms to share and discuss evidence-based findings that can promote a call to action for policy changes.^49^ These key contributions to health systems will support the delivery of holistic, patient-centered care services for TB patients.

Community fora can offer public platforms to share and discuss evidence-based findings that can promote a call to action for policy changes.

Health care workers should receive additional skills-based training in field epidemiology with case studies for outbreak investigations and operational research for TB prevention and control. This training can offer skills in identifying priority health issues, literature reviews, connecting with community stakeholders, data collection and analysis, and scientific writing.^39^^,^^41^^,^^49^ However, with increasing clinical and community health responsibilities—including allocation of staff and resources to new disease priorities—it is important to monitor and evaluate health care workers for mental health stressors and mitigate risk of burnout.^39^^,^^41^

#### Using Digital Health Interventions for TB Care

Existing electronic health technologies (eHealth), defined as platforms that use information and communication technologies,^52^ have the potential to change the paradigm in TB management. These digital innovations can offer remote support through video-supported therapy and electronic medication monitors for health care workers to guide TB patients through their clinical management, identify and monitor co-morbidities, and encourage treatment adherence.^33^^,^^36^^,^^38^^,^^39^ Mobile health solutions (mHealth) facilitate the delivery of short message service (SMS) and WhatsApp messages as well as geographic information systems (GIS) mapping for direct contact and delivery of test results and health information,^39^^,^^47^ especially reaching at-risk communities.^33^ With increased public interest observed during the COVID-19 pandemic, these digital approaches can enhance public engagement on infectious disease monitoring and offer informative fact sheets on multiple infectious diseases.^39^^,^^44^ These telemedicine applications, albeit with clear benefits for provider-patient engagement, raise potential questions for health care delivery. Some remaining issues include the ability to ensure patient safety, warrant data privacy and storage, conduct appropriate virtual physical exams, follow limited established protocols, evaluate cost-effectiveness, and assess adherence to improve overall equity and efficiency.^41^^,^^43^^,^^45^ Although comprehensive in-person evaluations cannot be eliminated from acute and chronic patient care,^42^ an integrated approach of in-person consultations and complementary telemedicine applications can pave the future for TB prevention and control efforts.

## DISCUSSION

This is the first known rapid review to examine the existing challenges to be addressed and proposed recommendations to strengthen TB efforts in low- and middle-income countries during and after the COVID-19 pandemic. The End TB Strategy targets were approached in 2020, but not met, with established targets of 20% decrease in incidence rates (reported 9% decrease) and 35% decrease in mortality rates (reported 14% decrease), with continued challenges in the Americas and African regions.^1^^,^^53^^,^^54^ Increased attention to TB efforts will be fundamental in the upcoming years to combat the decreased TB notification rates reported during the COVID-19 pandemic; support national policies that prioritize integral, patient-centered TB care; and expand current initiatives to end TB transmission.^53^

Increased attention to TB efforts will be fundamental in the future to combat the decreased TB notification rates reported during the COVID-19 pandemic, support national policies that prioritize patient-centered TB care, and expand current initiatives to end TB transmission.

Since emerging One Health threats continue to affect the global society, health leaders must be innovative in their contributions to clinical and community settings. Global health systems must be vigilant and prepared to adapt current evidence-based practices to meet societal needs of the next decade.^55^ In this review, selected studies identified challenges in TB control efforts—such as overwhelmed health systems and insufficient support for the health care workforce—that will require novel solutions to improve access and availability of diagnostic and treatment services for TB patients. The use of ground-breaking approaches, data, and practices that can help identify specific challenges, such as country-specific social, economic, and cultural contexts that influence health-seeking behaviors and treatment adherence,^35^ should be considered in maintaining sustainable TB programs.

A robust national health system with skilled leadership, trained health care workforce, health system and research investment, and direct connections with community stakeholders has the potential to achieve the national and international objectives of the Sustainable Development Goals (SDG 3.3) and the End TB Strategy. Political commitment will enhance national preparedness and guarantee an immediate public health response, noting stressors (e.g., dual health priorities) to a well-functioning health system.^56^ Continued investment in operational TB research is essential to identify knowledge gaps, develop sophisticated scientific inquiries, establish methodological and analytical approaches, and implement findings in public health practice.^54^^,^^57^^,^^58^

Strengthening links with primary care centers and relevant community stakeholders will elucidate local needs and resources as well as knowledge gaps related to TB care. National programs that advocate for public welfare and social protection for TB patients, including cash transfers, food parcels, housing resources, and psychosocial support, can minimize existing inequities in TB care, alleviate additional psychological stress, and offer indispensable support to TB patients and their families.^45^^,^^59^ Partnerships that expand public sector engagement with the private sector (public-private mix) for TB care are essential to meet the targets of the End TB Strategy, including ensuring prompt TB diagnosis and treatment, reducing hardships associated with catastrophic health care costs, and ultimately curbing TB transmission.^61^^,^^62^ National health statistics from active surveillance programs provide data on population health risks to authorities for health decision-making activities. Through qualitative research designs, health researchers can capture barriers related to TB treatment adherence, primary care providers' perspectives on TB control, and social determinants of health.^27^^,^^62^^,^^63^ Further exploration of the role and acceptance of digital innovations to TB care can be examined among the health care workforce and community members.^64^

Supporting a highly trained health care workforce and safe workplace environments is the responsibility of national health systems, especially with the renewed emphasis on the public-private mix for TB care and prevention. Comprehensive training with required annual continuing education hours for the health care workforce across the public and private sector will reinforce robust technical capacity, offer uniformity across institutions, and expand reach to resource-limited areas.^65^ Appropriate incentives for health care workers, such as financial bonuses, professional recognition, and supervisory positions, have the potential to motivate self-confidence and morale in professional competencies as well as enhance work satisfaction and productivity.^66^ Without this support, the brain drain phenomenon can lead to significant health care workforce shortages as workers migrate to other countries that offer improved working conditions, higher salaries or incentives, personal security, and professional recognition.^67^ Supervisors can also provide mentorship and objective feedback, encouraging health care workers to reach specific milestones in their performance plan.^68^

Digital health interventions can transform TB care by disseminating essential health information, supporting treatment adherence, and encouraging health-seeking behaviors among TB patients. With increased interest and versatility among health professional students, social media platforms have been successfully used to expand health communication efforts during the Ebola virus disease outbreak in West Africa^69^ and guide One Health community field campaigns.^70^ It has the potential to combat the “infodemic”—defined as the rapid spread of false information—of TB and other stigma-associated diseases like COVID-19^71^ as well as streamline operational networks in the public-private mix approach.^61^ Furthermore, telemedicine applications have strengthened provider-patient engagement and TB diagnostic and treatment adherence through text messaging^72^ and computer-aided technology.^73^ Patient safety issues, however, should be evaluated, especially ensuring data security and storage, confirming internet connectivity and related infrastructure, and training health workers in remote technologies.^43^^,^^73^ Although the use of broadband internet can substantially enhance the innovative delivery of TB health care services and educational programs, national health systems should identify existing disparities in internet access that can further exacerbate the “digital divide” as a social determinant of health.^11^^,^^74^

### Limitations

This study has a few limitations. First, although the authors participated in the data analysis and selected the final themes, they recognize that data may be interpreted in alternative ways.^75^ Second, the study utilized 5 databases for peer-reviewed articles but may have overlooked other relevant papers in the gray literature or other scientific databases. Finally, this study did not evaluate the quality of described challenges and recommendations of selected articles, although this task is not obligatory for rapid reviews.^24^ However, authors did critically analyze the study findings from original research articles and reviews with more substantial detail, than the letters and perspective articles.

## CONCLUSION

This rapid review summarizes areas for improvement in health system preparedness for optimal TB control across low- and middle-income countries. These findings can aid in the development of national policies to promote integral, patient-centered TB care, facilitate the implementation of ethical community interventions, support operational research, and allow the integration of appropriate eHealth applications. By better understanding challenges in TB prevention and control with coexisting health priorities, TB program managers and primary care practitioners can serve as instrumental leaders and patient advocates to deliver high-quality and sustainable TB care that leads to achieving targets of the End TB Strategy.

## Supplementary Material

GHSP-D-21-00368-supplement.pdf

## Notes

**Peer Reviewed**

**First published online:** November 30, 2021.
