# Supplementary material for: Lessons Learned During the COVID-19 Pandemic to Strengthen TB Infection Control: A Rapid Review
Source: Glob Health Sci Pract. 2021 Dec 31;9(4):964–77. doi: 10.9745/GHSP-D-21-00368 (PMC8691887; doi:10.9745/GHSP-D-21-00368)
Supplement: GHSP-D-21-00368-supplement.pdf [file GHSP-D-21-00368-supplement.pdf]

Supplement to: Chapman HJ, Veras-Estévez BA. Lessons learned during the COVID-19 pandemic to strengthen TB infection control: a scoping review. *Glob Health Sci Pract*. 2021;9(4). https://doi.org/10.9745/GHSP-D-21-00368

Supplement Table. Selected Articles for the Rapid Review of Literature on Recommendations for TB Prevention and Control During the COVID-19 Pandemic in Low- and Middle-Income Countries

Authors	Country or Continent	Article Type	Objective	Recommendations
Togun et al.[6]	UK, Africa	Review	To describe and analyze how the COVID-19 pandemic can impact TB control programs, using the UK and Africa as case studies	Noting differences between low- and high-income countries in TB priorities (active vs latent TB), provision of health services, and mechanisms for social protection, the global response should be comprehensive and long-term, increasing investments in research, innovative digital technology, and public health.
Hogan et al.[19]	Global	Original Research	To estimate the extent to which disruptions in HIV, TB, and malaria care in high-burden LMICs could influence mortality rates over five years	The most significant impact to increased mortality was the interruption to antiretroviral therapy for HIV, reductions in timely diagnosis and treatment of new cases for TB, and the interruption of planned net campaigns for malaria.
Visca et al.[20]	Global	Editorial	To describe the quality of life and the impact of digital technologies for TB during the COVID-19 pandemic	With expected increases in TB incidence and mortality, ensuring high levels of adherence to TB treatment through digital innovation can minimize the burden on patients and the health workforce.
Adamu et al.[31]	Africa	Commentary	To describe the need for a more holistic health systems-based model for the COVID-19 outbreak response in Africa	COVID-19 response strategies should shift from isolated programs to integrated health system interventions that are connected with existing programs in public and private sectors.
Alene, Wangdi & Clements[32]	Global	Viewpoint	To offer an overview of the potential impact of COVID-19 on the TB disease burden and highlight potential strategies to reduce the impact	Health systems should aim to maintain routine TB services during the COVID-19 pandemic, and hence mitigate the impact of COVID-19 on TB prevention and control programs.
Amimo, Lambert & Magit[33]	Africa	Short Report	To examine the potential implications of COVID-19 on the control of HIV, TB, and malaria in Africa	Appropriate economic and epidemiological considerations are required to minimize hardships faced by vulnerable populations to access essential health services for COVID-19 and other epidemic diseases.
Bhargava & Shewade[34]	India	Review Article	To describe how the lockdown impacts on poverty, nutrition and TB case detection can negatively influence TB	Federal support is required to improve economic and nutrition livelihood through cash transfers, public distribution system of food, and high-quality community TB surveillance and clinical management.

Supplement to: Chapman HJ, Veras-Estévez BA. Lessons learned during the COVID-19 pandemic to strengthen TB infection control: a scoping review. *Glob Health Sci Pract*. 2021;9(4). https://doi.org/10.9745/GHSP-D-21-00368

Authors	Country or Continent	Article Type	Objective	Recommendations
			incidence and mortality in India	
Bulled & Singer[35]	South Africa	Commentary	To examine the public health response to the COVID-19 pandemic, how the HIV/TB epidemics influence the COVID-19 response, and implications of the response in South Africa	International cooperation and country-specific efforts that reflect local resources and needs are required to overcome significant public health risks by the COVID-19 pandemic.
Dara et al.[36]	Global	Letter	To describe lessons from TB control for the COVID-19 response	Complementary COVID-19 and TB responses – including capacity building, active surveillance and monitoring systems, and sustainable economic investment – have the potential to curb disease transmission.<br>People-centered care models, including video-supported or home-based care, community-based services, and hospitalization can be adapted for the COVID-19 and TB epidemics.
Homolka et al.[37]	Global	Perspective	To analyze the potential use of TB diagnostic and research infrastructures for SARS-CoV-2 testing	TB diagnostic and research infrastructures can be leveraged for SARS-CoV-2 testing and sequencing to examine virus evolution and diversity.<br>High-quality management principles for TB and SARS-CoV-2 diagnostic testing must be followed to ensure validity, reduce biosafety hazards, and support TB diagnostic services.
Jain et al.[38]	India	Review	To describe the challenges and potential solutions in TB management due to the COVID-19 pandemic in India	Restructuring services – such as multi-month dispensing, video-supported therapy, and community-based care – can strengthen TB programs.
Loveday et al.[39]	South Africa	Review	To describe the impact of the COVID-19 pandemic and lockdown on TB control in South Africa	Integrating COVID-19 systems to support TB prevention and control can include developing a platform for public engagement on disease monitoring, strengthening contact tracing with GIS mapping, offering mobile- or video-supported counseling and clinical management, improving health information and surveillance systems, and increasing federal investment.
McQuaid et al.[40]	Global	Letter	To describe the potential impact of COVID-19 disruption effects on TB burden	To ensure continued access to person-centered TB care, sustainable funding, innovative digital technology, and robust community-based surveillance activities,

Supplement to: Chapman HJ, Veras-Estévez BA. Lessons learned during the COVID-19 pandemic to strengthen TB infection control: a scoping review. *Glob Health Sci Pract*. 2021;9(4). https://doi.org/10.9745/GHSP-D-21-00368

Authors	Country or Continent	Article Type	Objective	Recommendations
				which can be expanded to reduce the TB and COVID-19 burden.
Mohammed et al.[41]	Ethiopia	Commentary	To examine the COVID-19 lockdown measures and recommend actions to leverage the health system response to COVID-19 and TB in Ethiopia	Continued investment in TB care and research activities is key to minimize disruptions to health and research services.
Mukwenha et al.[42]	Zimbabwe	Perspective	To describe the public health dilemma of sustained HIV/TB services during the COVID-19 pandemic in Zimbabwe	Through collaborations with local and international partners, Zimbabwe leaders can strengthen HIV/TB services by ensuring stockpile availability of diagnostic testing, disseminating accurate health information to TB patients, and adopting real-time surveillance systems.
Papadimos et al.[43]	Global	Consensus paper	To provide a high-level synopsis of public health "blind spots" observed during the COVID-19 pandemic	Deploying point-of-care diagnostics and focusing on telemedicine platforms (albeit challenges like suboptimal internet connectivity or insufficient encryption) have the potential to enhance screening efforts and prevent excess TB mortality.
Sandy et al.[44]	Zimbabwe	Letter	To describe the high HIV/TB burden in Zimbabwe re-	Urgent responses include increased funding for equipment (PPE, sputum containers), monthly medication supplies, and integrated HIV/TB programs that distribute appropriate health information.
Saunders and Evans[45]	Global	Editorial	To describe how the COVID-19 pandemic can impact TB prevention and control efforts	Integrated health care for TB and COVID-19, research investment, community mobilization, TB-specific social protection, and innovative digital technologies can strengthen TB control efforts during the COVID-19 pandemic.
Zachariah et al.[46]	Global	Original research	To examine the contributions of health workers (trained through Structured Operational Research and Training IniTiative, SORT IT) during the COVID-19 response and identify their application of SORT IT skills	Skill-building trainings can support outbreak responses (from data collection to scientific writing) and surveillance programs.<br>Investments in health research can strengthen health system resiliency with robust surveillance programs and a prepared workforce.
Zhou, Van Staden and Toska[47]	South Africa	Letter	To describe resource reprioritization and competing health risks for the TB and COVID-	Services implemented during the COVID-19 pandemic, such as GIS mapping, can be repurposed to strengthen TB control efforts.

Supplement to: Chapman HJ, Veras-Estévez BA. Lessons learned during the COVID-19 pandemic to strengthen TB infection control: a scoping review. *Glob Health Sci Pract*. 2021;9(4). https://doi.org/10.9745/GHSP-D-21-00368

Authors	Country or Continent	Article Type	Objective	Recommendations
			19 response in South Africa	Reliable health services to care for TB and COVID-19 patients are key to reducing stigma and building trust in health systems.
